# The complete mitochondrial genome sequence of *Dodona eugenes* (Lepidoptera: Riodinidae)

**DOI:** 10.1080/23802359.2021.1884014

**Published:** 2021-03-11

**Authors:** Zhong-Xue Wei, Gang Sun, Jer-Yuan Shiu, Yan Fang, Qing-Hui Shi

**Affiliations:** a Medical Plant Exploitation and Utilization Engineering Research Center, Sanming University, Sanming, PR China; b Fujian Provincial Key Laboratory of Resources and Environment Monitoring & Sustainable Management and Utilization, Sanming University, Sanming, PR China

**Keywords:** Mitochondrial genome, Riodinidae, *Dodona eugenes*, phylogenetic analysis

## Abstract

The complete mitochondrial genome (mitogenome) sequence of *Dodona eugenes* (Lepidoptera: Riodinidae) was determined and analyzed. The mitogenome is 15,680 bp in length with consisting of 13 protein-coding genes (PCGs), 22 transfer RNA (*tRNA*) genes, two ribosomal *RNA* genes (*rrnL* and *rrnS*), and one AT-rich region. The gene content, orientation, and order are identical to that of the majority of other lepidopteran insects. The *D. eugenes* mitogenome includes a *cox1* gene with an atypical CGA(R) start codon and three genes (*cox1*, *nad5*, and *nad4*) exhibiting incomplete stop codons. All tRNAs have a typical secondary cloverleaf structure, except for *trnS1* which lacks the dihydrouridine (DHU) arm. The 825-bp long AT-rich region is the longest among sequenced riodinids, which range from 349 to 423 bp. The conclusion of phylogenetic analysis highly supported the monophyly of Riodinidae, which is standing as the sister of the family Lycaenidae.

The insect mitochondrial genomes (mitogenomes) provide effective data for studies on systematic, population genetics, and evolutionary biology (Cameron 2014; Yang et al. 2020). But for Riodinidae, a pantropical family of butterflies with the majority (93%) of species occurring in the neotropics (DeVries 1997; Espeland et al. 2015), only three complete mitogenomes are available now (Zhao et al. 2013; Kim and Kim 2014; Shi et al. 2020).

For better understanding of the phylogenetic position and higher systematics of riodinids, the complete mitogenome of *Dodona eugenes* needs to be determined. The specimen was collected from Sanming in Fujian Province, China (coordinates: E117°62′, N26°27′), and kept in the laboratory at −20 °C under the accession number SQH-20170628. Total genomic DNA was extracted from thorax muscle of an adult individual using the Rapid Animal Genomic DNA Isolation Kit (Sangon, Shanghai, China). The raw sequences were assembled and annotated using the BioEdit version 7.0 (Hall 1999) and MEGA version 7.0 software (Kumar et al. 2016) with reference to the mitogenome of *Abisara fylloides* (GenBank accession no. HQ259069).

The complete mitogenome of *D. eugenes* contained 13 protein-coding genes (PCGs), 22 transfer RNA (*tRNA*) genes, two ribosomal RNA (*rRNA*) genes, and one AT-rich region, with the sizes of 15,680 bp (GenBank accession no. MT890732). Its gene content and arrangement are similar to those of other butterflies (Zhao et al. 2013; Wu et al. 2014). The nucleotide composition is significantly A + T biased (81.0%). Besides the AT-rich region, 12 intergenic spacers (143 bp in total) and 10 overlapping regions (63 bp in total) are dispersed throughout the whole genome.

The concatenated PCGs are 11,208 bp long accounting for approximately 71.5% of the mitogenome. All PCGs are initiated by typical ATN, with the exception of *cox1* which uses the unusual CGA(R) as observed in most other sequenced butterflies (Kim and Kim 2014; Wu et al. 2014). Ten PCGs have canonical termination codons TAN, while three (*cox1*, *nad5*, and *nad4*) have incomplete termination codons single T. All tRNAs exhibit typical cloverleaf secondary structures, except for *trnS1*(*AGN*), which lacks the dihydrouridine (DHU) arm, as universally found in other butterfly mitogenomes. The length of *rrnL* and *rrnS* are 1325 and 771 bp, respectively, separated by *trnV*. The 825 bp long AT-rich region is longer than other sequenced riodinids, including several structures characteristic of lepidopterans, such as the ATAGA motif followed by a poly-T stretch, a microsatellite-like element preceded by the ATTTA motif (Kim et al. 2014; Salvato et al. 2008).

Phylogenetic tree was reconstructed by MrBayes version 3.1.2 (Ronquist and Huelsenbeck 2003) based on concatenated nucleotide sequences of 13 PCGs and 2 rRNAs from *D. eugenes* and other 47 representatives from six families and two outgroup species (see Figure 1 for details). The phylogenetic analysis revealed that Riodinidae is a single family being the sister group to Lycaenidae. These results are all consistent with previous studies (Shen et al. 2015; Espeland et al. 2018). However, more taxa and mitogenomes are needed in order to clarify the phylogenetic relationships within Riodinidae in the future.

**Figure 1.**
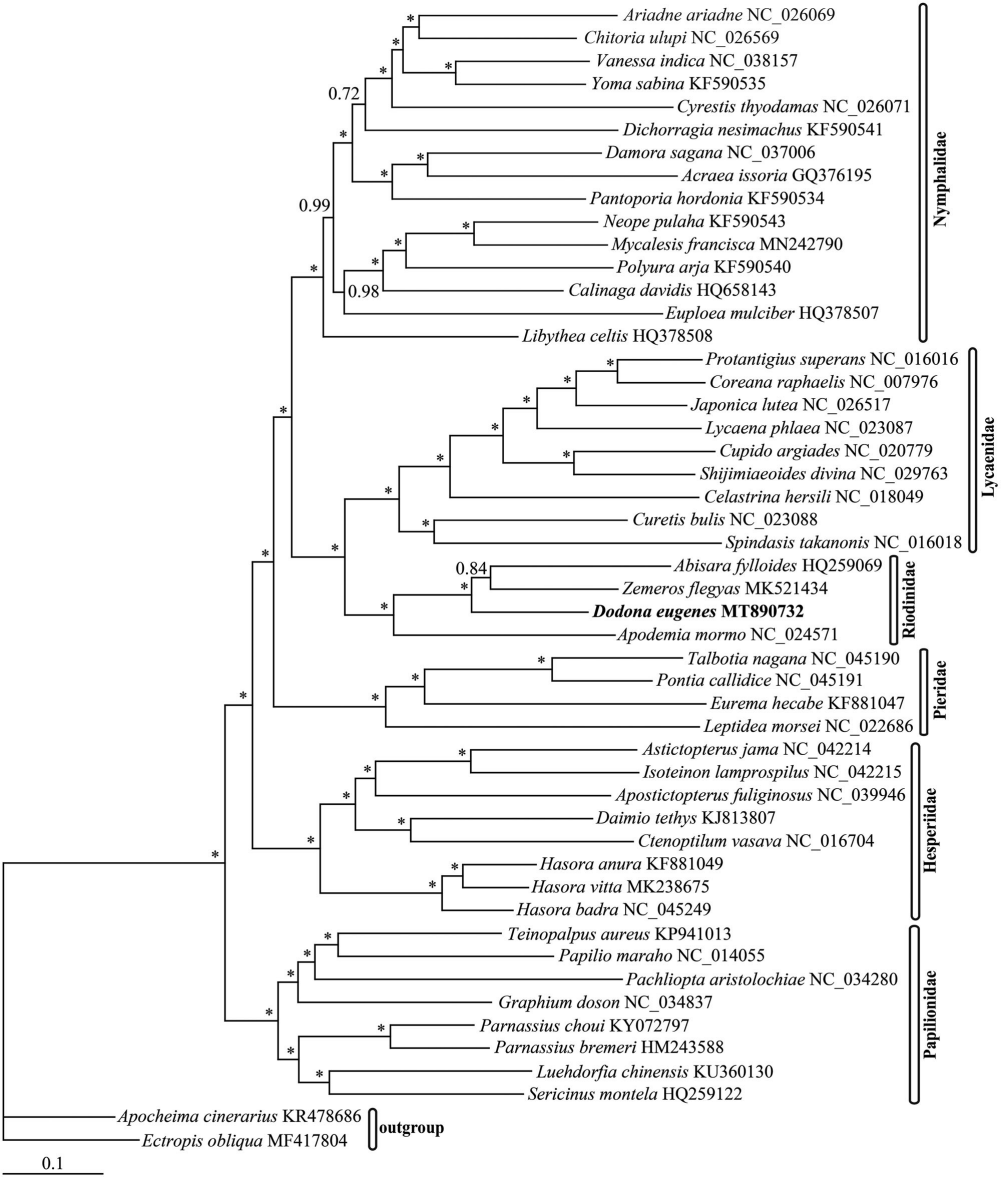
The Bayesian inference (BI) phylogenetic tree of *Dodona eugenes* and other butterflies. Phylogenetic reconstruction was done from a concatenated matrix of 13 protein-coding mitochondrial genes and two ribosomal *RNA* genes. The numbers beside the nodes correspond to the posterior probability values (* = 1.00). Alphanumeric terms indicate the GenBank accession numbers.

## Data Availability

The data that support the findings of this study are openly available in GenBank (accession no. MT890732) at https://www.ncbi.nlm.nih.gov/genbank/, moreover, the sequence data reported in this paper has been submitted to Baidu Netdisk at https://pan.baidu.com/s/1-mE4i9o9LcJcpSGirYjxQg (extraction code: 6qnm).
